# GAS6-based CAR-T cells exhibit potent antitumor activity against pancreatic cancer

**DOI:** 10.1186/s13045-023-01467-9

**Published:** 2023-07-20

**Authors:** Jiawei Fan, Ye Yu, Lanzhen Yan, Yuncang Yuan, Bin Sun, Dong Yang, Nan Liu, Jing Guo, Jie Zhang, Xudong Zhao

**Affiliations:** 1 Division of Abdominal Tumor Multimodality Treatment and Laboratory of Animal Tumor Models, Cancer Center and State Key Laboratory of Biotherapy and National Clinical Research Center for Geriatrics and Frontiers Science Center for Disease-Related Molecular Network, West China Hospital, Sichuan University, Chengdu, 610041 Sichuan China; 2 Core Facilities of West China Hospital, Sichuan University, Chengdu, 610041 Sichuan China

**Keywords:** CAR-T-cell therapy, TAM family, GAS6, Pancreatic cancer

## Abstract

**Background:**

The receptor tyrosine kinases TAM family (TYRO3, AXL, and MERTK) are highly expressed in multiple forms of cancer cells and tumor-associated macrophages and promote the development of cancers including pancreatic tumor. Targeting TAM receptors could be a promising therapeutic option.

**Methods:**

We designed a novel CAR based on the extracellular domain of growth arrest-specific protein 6 (GAS6), a natural ligand for all TAM members. The ability of CAR-T to kill pancreatic cancer cells is tested in vitro and in vivo, and the safety is evaluated in mice and nonhuman primate.

**Results:**

GAS6-CAR-T cells efficiently kill TAM-positive pancreatic cancer cell lines, gemcitabine-resistant cancer cells, and cancer stem-like cells in vitro. GAS6-CAR-T cells also significantly suppressed the growth of PANC1 xenografts and patient-derived xenografts in mice. Furthermore, these CAR-T cells did not induce obvious side effects in nonhuman primate or mice although the CAR was demonstrated to recognize mouse TAM.

**Conclusions:**

Our findings indicate that GAS6-CAR-T-cell therapy may be effective for pancreatic cancers with low toxicity.

**Supplementary Information:**

The online version contains supplementary material available at 10.1186/s13045-023-01467-9.

## Introduction

Pancreatic cancer is the most lethal malignancy with less than 10% of patients surviving five years after diagnosis [1]. In terms of new treatments, chimeric antigen receptor T (CAR-T) cell therapies have recently been shown to be highly successful for hematological malignancies, and this approach also shows promising results against solid tumors, including pancreatic cancers. However, current CAR-T cells targeting a range of different proteins have not shown remarkable efficacy against pancreatic cancer in clinical trials [2], indicating the need to explore more effective CAR-T strategies.

Receptor tyrosine kinase TAM (TYRO3, AXL, MERTK) family members are overexpressed in several hematological malignancies, including acute myeloid leukemia, chronic myeloid leukemia, and acute lymphoid leukemia [3, 4], and in different types of solid tumors, such as pancreatic, lung, gastric, and breast cancers [5]. TAM family promotes tumor cell proliferation, invasion, metastasis, drug resistance, and immune escape, and their expression is negatively correlated with prognosis in cancer patients [3].

TAM receptors have emerged as promising therapeutic targets. For example, BGB324, a small molecule inhibitor of AXL, has entered phase I/II clinical trials for acute myeloid leukemia and pancreatic cancer [6]. Monoclonal anti-AXL antibodies could suppress the growth and metastasis of variety of cancers [6–8]. AVB-500, a high-affinity AXL fusion protein, effectively increasing the chemosensitivity of ovarian cancer and endometrial cancer [9, 10], is currently being tested in a Phase Ib clinical trial against platinum-resistant ovarian cancer [11]. Anti-TYRO3 antibodies inhibited the cancer progression or metastasis of colon cancer and melanoma cells [12, 13]. MERTK monoclonal antibodies promoted the apoptosis of triple-negative breast cancer and non-small cell lung cancer [14, 15]. Targeting TAM receptors has also been an effective method to re-sensitize resistant cells [16]. However, AXL inhibition may lead to up-regulation of other TAM members such as MERTK, which is linked to acquired drug resistance in preclinical models of head and neck squamous cell carcinoma, triple-negative breast cancer, and non-small cell lung cancer, and combination therapy targeting both AXL and MERTK could eliminate the acquired resistance and inhibit tumor growth [17]. Therefore, targeting multiple TAM members may provide an effective means of preventing cancer drug resistance.

CAR-T is an emerging therapy targeting membrane proteins, and this approach has seen success in hematological malignancy. AXL-directed CAR-T cells have proven to be effective at inhibiting the growth of triple-negative breast cancer and chronic myelogenous leukemia [18, 19]. The growth arrest-specific protein 6 (GAS6) is a natural ligand for all TAM family members with the highest affinity for AXL [3]. In this study, we generate CAR-T cells based on GAS6 and demonstrate that these GAS6-CAR-T cells can recognize all the TAM members, efficiently kill pancreatic cancer cells, and inhibit the growth of tumor xenografts without causing any overt side effects in mice even when the CAR-T is demonstrated to recognize mouse TAM and efficiently lyse mouse tumor cells.

## Materials and methods

### Cell lines

The human pancreatic cancer cell lines ASPC1, BxPC3 and PANC1, human embryonic kidney 293 T cells (HEK-293 T), mouse embryonic fibroblast cell line NIH 3T3, and mouse breast cancer cell line 4 T-1 used in this study were maintained in our laboratory. We obtained the human pancreatic cancer cell line MIA PaCa2 and mouse hepatoma carcinoma cell line Hepa1-6 from the cell bank of the Chinese Academy of Sciences (Shanghai, China). The ASPC1-gemcitabine-resistant cell line was purchased from FENGHUISHENGWU Co. Ltd. (Hunan, China). All cell lines were authenticated by STR, and mycoplasma contamination was routinely tested by qPCR.

Luciferase-labeled cells were established by infection with pTomo-CMV-luciferase-IRES-Puro lentivirus followed by selection with puromycin (1.0 mg/mL, Gibco, USA) for 2 weeks. All cells were cultured in DMEM containing 10% fetal bovine serum (Gibco, USA), 100U/mL penicillin, and 100 mg/mL streptomycin (Gibco, USA). The culture medium of Hepa1-6 cells also contained 1.0 mM sodium pyruvate (Gibco, USA) and Gluta-MAX™ (100× , Gibco, USA), and gemcitabine-resistant ASPC1 cells were cultured with 1.0 ug/mL gemcitabine. Suspended cell spheres derived from PANC1 and MIA PaCa2, named PANC1-CSC and MIA PaCa2-CSC, respectively, were established by culturing in serum-free stem cell medium composed of DMEM/F12 (Gibco, USA), EGF (20 ng/mL, PeproTech., USA), bFGF (20 ng/mL, PeproTech., USA), and B27 (1× , Gibco, USA).

### Plasmid construction and lentiviral production

CAR comprising the CD8 signal peptide, extracellular domain (amino acids 261–678) of human GAS6, the CD8 hinge spacer and transmembrane domain, CD137 (4-1BB), and the CD3ζ endo domains was cloned into the pTomo-Puro plasmid (Addgene, USA) between the AgeI and NheI restriction sites. An mKATE2 sequence was fused to the CAR via a T2A peptide to monitor the transduction efficiency. The same vector sequence without extracellular domain of GAS6 was used as a control (Mock).

To construct TAM-shRNA plasmids, the target sequences were cloned into a pLKO.1-Puro vector obtained from Addgene between the AgeI and EcoRI restriction sites. The target sequences were as follows: shAXL #1, CGAAATCCTCTATGTCAACAT, #2, CGAAAGAAGGAGACCCGTTAT; shTYRO3 #1, GGAGAGGAACTACGAAGAT CG, #2, GCATCAGCGATGAACTAAAGG; shMERTK #1, GCTCAATCAGTGTAC CTAATA, #2, GCATTGGTGTTTCCTGCATGA. Expression plasmids containing AXL (EX-Z7835-Lv105), TYRO3 (EX-A0969-Lv105), and MERTK (EX-Z8208-Lv105) were purchased from iGene Biotechnology Co., Ltd. (Guangzhou, China).

For lentiviral packaging, plasmids were transfected into HEK-293 T cells with the packaging plasmids pCMV-dR8.91 and pMD 2.G (Addgene) at a ratio of 5:2.5:1. The supernatants were collected and filtered through a 0.45-μm filter (Millipore, Bedford, MA) to remove cellular debris and centrifuged at 25,000 rpm for 2.5 h to obtain the virus precipitation.

### Production of CAR-T cells

Human T cells were isolated from healthy donor blood using the RosetteSep™ Human T-Cell Enrichment Cocktail (STEMCELL, Canada) and cultured in advanced 1640 medium (Gibco, USA) containing 10% FBS (Gibco, USA) with 200 U/mL IL-2 (Invitrogen, USA) and Gluta-MAX™ (100× , Gibco, USA). To generate CAR-T cells, T cells were activated by CD3/CD28 dynabeads (Life Technologies, USA) for 72 h followed by incubation with lentiviral particles at an approximate MOI of 100 with lentiBoost (1.0 μg/mL, Sirion Biotech, Germany) for 24 h. The CAR-T cells were applied for experiments on day 3 after transduction.

Monkey T cells isolated from rhesus monkeys by density gradient centrifugation (Ficoll-Paque) were activated by nonhuman primate T-cell activation/expansion kit (Miltenyi Biotec) and cultured in RPMI-1640 medium containing 10% FBS with 200 U/mL IL-2 and Gluta-MAX™. The activated T cells were transduced with lentiviral particles of GAS6-CAR to prepare CAR-T cells.

### In vitro cytotoxicity assays

The cytotoxicity of CAR-T cells was tested using a Luciferase Assay System (Promega, E1501) at variable effector-to-target (E/T) ratios of 0.5:1, 1:1, 2:1, and 4:1. Briefly, 2 × 10^3^ target cells per well were seeded in 96-well plates with 100 μL medium, and an equal volume of effector cells was added. After 24 h of coculture, the supernatant was collected and used to determine the concentrations of IFN-γ (Invitrogen, KHC4021) and TNF-α (Proteintech, KE00154). The cells were then lysed for luciferase assay according to the instructions of the manufacturer, and the cytotoxicity of CAR-T cells was calculated as ratio to tumor cells incubated with non-transduced T (NT) cells. The results were expressed as means and standard deviations for triplicate assays.

### Western blot assays

Western blotting was performed as described previously [20]. Harvested cells were lysed in RIPA buffer, and protein concentrations were quantified using BCA protein assay kits (Beyotime, Shanghai, China). The total protein lysates were separated by 10% sodium dodecyl sulfate polyacrylamide gel electrophoresis and transferred to PVDF membranes (Millipore, Billerica, MA, USA). The membranes were blocked in Tris-buffered saline with 5% non-fat milk and 0.5% BSA for 1 h, prior to incubation with primary antibodies overnight at 4 °C and incubation with horseradish peroxidase (HRP)-conjugated secondary antibodies for 1.5 h at room temperature. Blots were visualized with chemiluminescent HRP substrate (Millipore). Detailed information of antibodies used in this experiment is listed in Additional file 1: Table S1.

### Flow cytometry

The cells (1 × 10^6^) were fixed in 4% formaldehyde for 15 min at room temperature. After washing, the cells were incubated with primary antibodies for 1 h and then the fluorescent secondary antibodies for 30 min at room temperature (Additional file 1: Table S1). Finally, the cells were analyzed by BD LSRFortessa Flow cytometry (BD Biosciences), and data were analyzed using FlowJo software version 10 (TreeStar, Inc.).

### Quantitative real-time PCR

Total RNA and genomic DNA were extracted as described previously [20, 21]. qPCR assays were performed with SYBR Selected Master Mix (Thermo Fisher, USA). The comparative cycle time (Ct) method was used to determine differences between samples, and the expression of target genes was normalized to 18S rRNA or GAPDH (2^−△△Ct^). The primer sequences are listed in Additional file 1: Table S2.

### Mouse models of cell-derived xenografts (CDX)

5 × 10^5^ PANC1-luciferase cells were suspended in PBS containing 20% Matrigel (BD Bioscience) and subcutaneously injected into the right flank of six-week-old female NOD/ShiLtJGpt-Prkdc^em26Cd52^Il2rg^em26Cd22^ /Gpt (NCG) mice (GemPharmatech Co. Ltd., China). Mice were intraperitoneally injected with 150 mg/kg D-luciferin (BioVison, 7903-1G) after anesthesia with 1.5% isoflurane, and tumor progression was determined using an in vivo imaging software (IVIS) system (Guangzhou Biolight Biotechnology Co., Ltd., aniview100). The mice were randomly divided into two groups according to bioluminescent signals at 3rd day and treated with 1 × 10^7^ Mock T cells or GAS6-CAR-T cells by tail vein injection. Bioluminescent signals were subsequently measured every 7 days.

### Patient-derived xenograft (PDX) model of pancreatic cancer

To establish the PDX model of pancreatic cancer, 3 × 3 mm blocks of patient-derived pancreatic tumor tissues were implanted in the right flank of six-week-old female NCG mice. After 14 days, the mice were randomly divided into two groups and treated with an injection of 1 × 10^7^ Mock T cells or GAS6-CAR-T cells. Tumor size was measured twice a week using a digital caliper, and tumor volume was calculated using the following formula: (major axis of tumor) × (minor axis of tumor)^2^/2. The mice were euthanized when the tumor volume reached 1000 mm^3^. This experiment was completed with the assistance of Sichuan Kang Cheng Biotechnology Co. (Chengdu, China).

### Immunohistochemistry

To detect multicolor immunofluorescence, we performed this experiment using Opal™ Multiplex IHC Assay (Akoya Biosciences, USA) that allowed to use any standard unlabeled primary antibody, including multiple antibodies raised in the same species.

Tissues were fixed with 4% paraformaldehyde, dehydrated with gradient ethanol, and embedded in paraffin. Tissues slides were dewaxed and dehydrated, boiled in citrate buffer (pH 6.0) for antigen retrieval, and blocked using 5% normal goat serum at room temperature for 1 h. Then, the slides were incubated at 4 °C overnight with the following primary antibodies and incubated for 1 h with the corresponding HRP-conjugated secondary antibodies (Additional file 1: Table S1) and TSA Plus Fluorescein Reagent (1:50) for 10 min. Finally, nuclei were stained with DAPI. Fluorescent images were taken using a confocal microscope (Nikon, Japan), and representative microscopy images were shown.

### Statistical analysis

Statistical analyses were performed using GraphPad Prism version 8.0 (GraphPad Software Inc.). All data are presented as mean ± SD. Statistical differences between two groups were analyzed using Student’s t tests with Welch correction. Statistical differences among three or more groups were analyzed by one-way or two-way ANOVA with Sidak correction. In all statistical analyses, the *P* values (*P < 0.05, **P < 0.01) were considered significant, ns = not significant.

## Results

### GAS6-CAR specifically recognizes the TAM family of receptor tyrosine kinase

Laminin g-like domain (LG domain) of GAS6 (amino acids 261–678) binds to the immunoglobulin-like domain of TAM receptors [3, 22, 23]. To target the TAM family, we used the LG domain of GAS6 as the recognition domain in CAR design. The CAR and control vector are graphically represented in Additional file 1: Fig. S1A. Flow cytometry analysis of mKATE2 fluorescence revealed a transduction efficiency of approximately 35% (Additional file 1: Fig. S1B, C). The lentiviral transduction and exogenous CAR expression had no significant effects on the proliferation of T cells (Additional file 1: Fig. S1D).

**Fig. 1:**
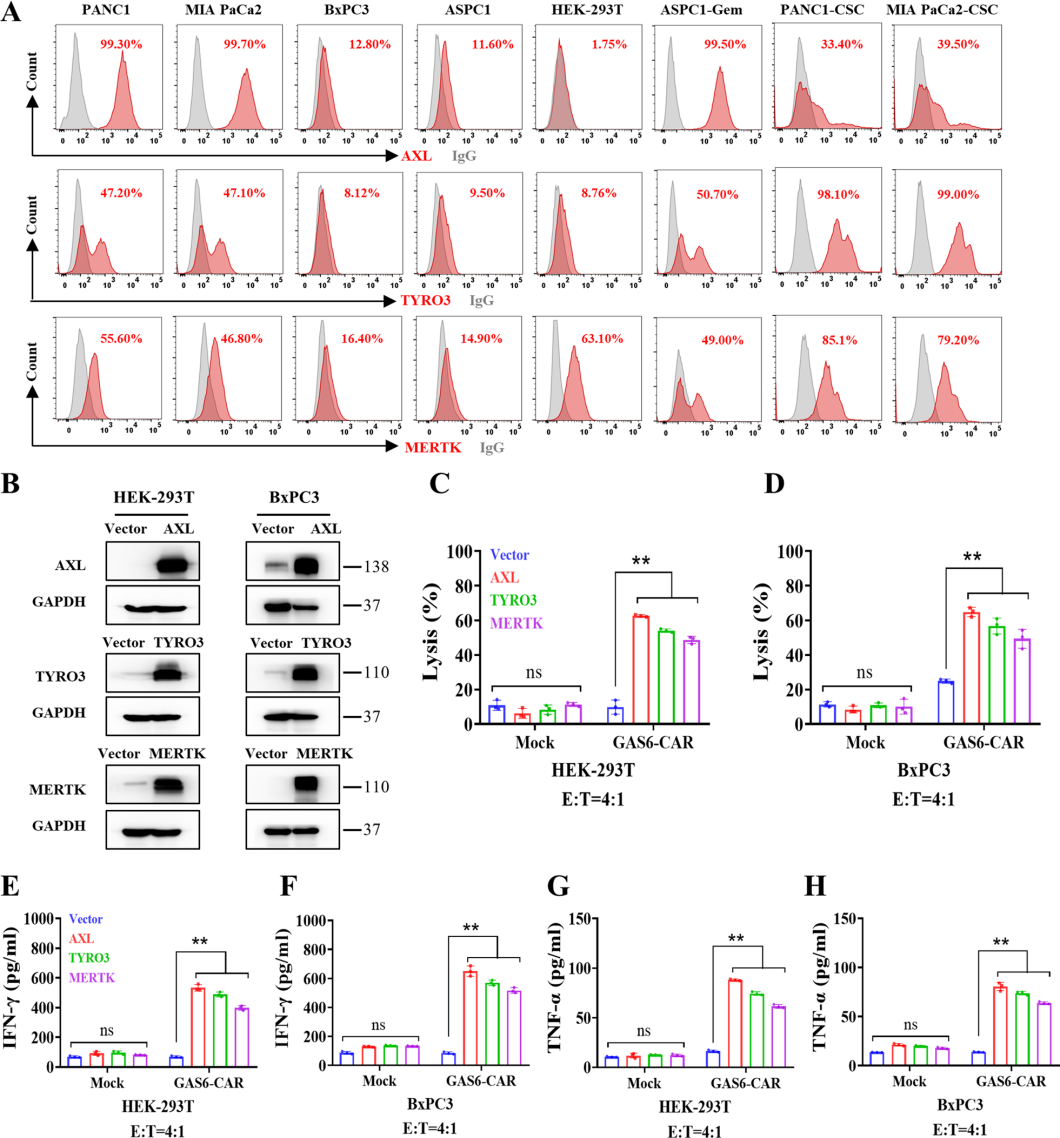
GAS6-CAR-T cells target cells overexpressing TAM members. **A** Flow cytometry analysis of TAM family (AXL, TYRO3, and MERTK) protein levels in various pancreatic cancer cell lines (PANC1, MIA PaCa2, BxPC3, and ASPC1), human embryonic kidney cell 293 T (HEK-293 T), ASPC1-gemcitabine-resistant (ASPC1-Gem) cells, and cell line-derived cancer stem cells (PANC1-CSC and MIA PaCa2-CSC). **B** The level of TAM protein overexpression in HEK-293 T and BxPC3 cell lines was tested by western blot, and GAPDH was used as a loading control. Cytotoxicity of GAS6-CAR-T cells on TAM-overexpressing HEK-293 T (**C**) and BxPC3 (**D**) cells at an E/T ratio of 4:1 for 24 h (*n* = 3). Enzyme-linked immunosorbent assay (ELISA) was used to analyze IFN-γ (**E**&** F**) and TNF-α (**G**&** H**) release by either Mock T cells or GAS6-CAR-T cells in coculture supernatant

We detected the expression of TAM proteins in pancreatic cancer cells. As shown in Fig. 1A, AXL, TYRO3, and MERTK were found to be highly expressed in PANC1 and MIA PaCa2 and low levels in BxPC3 and ASPC1, while human embryonic kidney cell line HEK-293 T expressed a relatively higher level of MERTK. To determine whether GAS6-CAR can target the TAM family members, AXL, TYRO3, and MERTK were individually transduced in TAM-low HEK-293 T and BxPC3 cells, and the overexpression was confirmed by western blot analysis (Fig. 1B). Luciferase-labeled AXL-, TYRO3-, or MERTK-overexpressing HEK-293 T and BxPC3 cells were co-incubated with CAR-T cells, and luciferase activity was measured after 24 h incubation. As shown in Fig. 1C, D, GAS6-CAR-T cells efficiently killed cells overexpressing any TAM protein, but had no effect on the control cells. The antigen-stimulated release of the IFN-γ (Fig. 1E, F) and TNF-α (Fig. 1G, H) cytokines was induced in these CAR-T cells. These data demonstrate that GAS6-CAR-T cells can target all three TAM proteins.

### GAS6-CAR-T cells specifically target TAM-positive pancreatic cancer cells

**Fig. 2:**
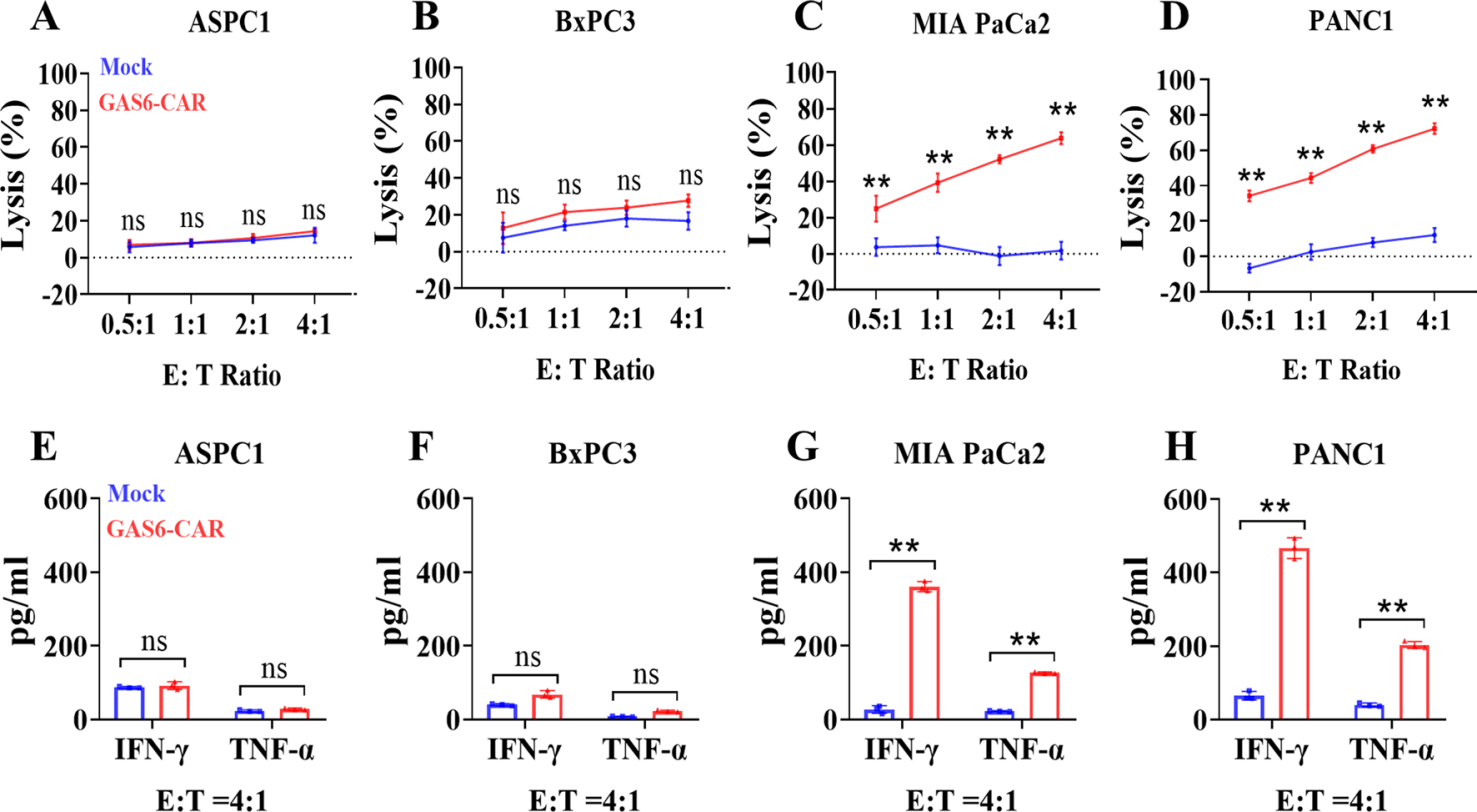
GAS6-CAR-T cells efficiently lyse TAM-positive human pancreatic cancer cell lines. The cytotoxicity of GAS6-CAR-T cells on TAM-low ASPC1 (**A**) and BxPC3 (**B**), TAM-high MIA PaCa2 (**C**), and PANC1 (**D**) cell lines was tested at varying effector-to-target (E/T) ratios for 24 h (*n* = 3). Quantification of IFN-γ and TNF-α release in response to coculture with Mock T cells or GAS6-CAR-T cells at an E/T ratio of 4:1 in ASPC1 (**E**), BxPC3 (**F**), MIA PaCa2 (**G**), and PANC1 (**H**), as measured by ELISA

We next assessed the capacity of GAS6-CAR-T cells to kill TAM-positive pancreatic cancer cells. Compared to Mock T cells, incubation with GAS6-CAR-T cells led to significantly higher rates of death in the TAM-positive MIA PaCa2 and PANC1 cells in a dose-dependent manner (Fig. 2C, D), but no killing effects on TAM-low ASPC1 and BxPC3 cell lines (Fig. 2A, B). The mRNA transcript levels for IFN-γ, TNF-α, IL-2, and IL-10 were significantly increased (Additional file 1: Fig. S2C, D), and the release of IFN-γ and TNF-α into the supernatant was also significantly increased in the incubation with TAM-positive MIA PaCa2 and PANC1 cells (Fig. 2G, H), while the expression of cytokines did not increase in incubation with TAM-low ASPC1 and BxPC3 cell lines (Fig. 2E, F and Additional file 1: S2A, B). Thus, the GAS6-CAR-T cells appear to specifically target TAM-positive pancreatic cancer cells.

**Fig. 3:**
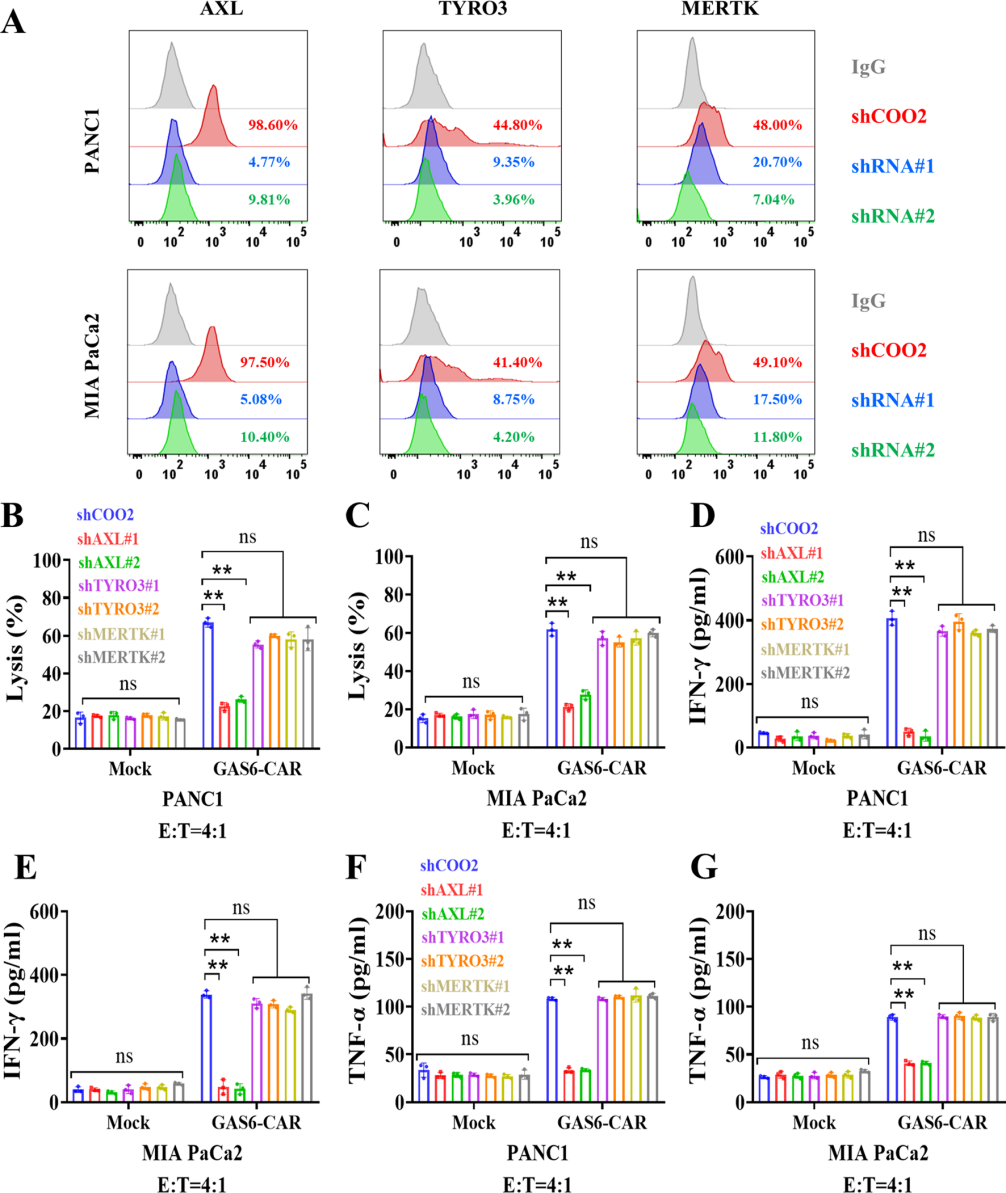
GAS6-CAR-T cells cytotoxicity is abolished by silencing AXL. **A** The knockdown efficiency of shRNAs targeting AXL, TYRO3, or MERTK was tested by flow cytometry. Cytotoxicity of GAS6-CAR-T cells on target cells PANC1-shRNA (**B**) and MIA PaCa2-shRNA (**C**) was tested at an E/T ratio of 4:1 for 24 h (*n* = 3). ELISA-based quantification of IFN-γ (**D**&** E**) and TNF-α (**F**&** G**) release in response to coculture with Mock T cells or GAS6-CAR-T cells

To determine which TAM member was responsible to CAR-T effects, we tested the effects of knocking down individual TAM proteins. We identified and confirmed the silencing efficiency of two shRNA sequences for each TAM protein (Fig. 3A). Both AXL-shRNAs significantly abolished the cytotoxic effects of GAS6-CAR-T cells toward PANC1 and MIA PaCa2 target cells (Fig. 3B). Furthermore, the antigen-specific expression of IFN-γ, TNF-α, IL-2, and IL-10 was significantly reduced (Additional file 1: Fig. S3), and the secretion of IFN-γ (Fig. 3D, E) and TNF-α (Fig. 3F, G) maintained at low level. However, shRNA sequences against TYRO3 or MERTK had no significant effects on the cytotoxicity of GAS6-CAR-T cells (Fig. 3B, C) and the secretion of cytokines (Fig. 3D–G). It suggests that AXL is the main target in tested cell lines, which is probably involved in the different expression of TAM members and highest affinity of AXL to GAS6.

### GAS6-CAR-T cells are effective against drug-resistant cell lines and cancer stem-like cells

**Fig. 4:**
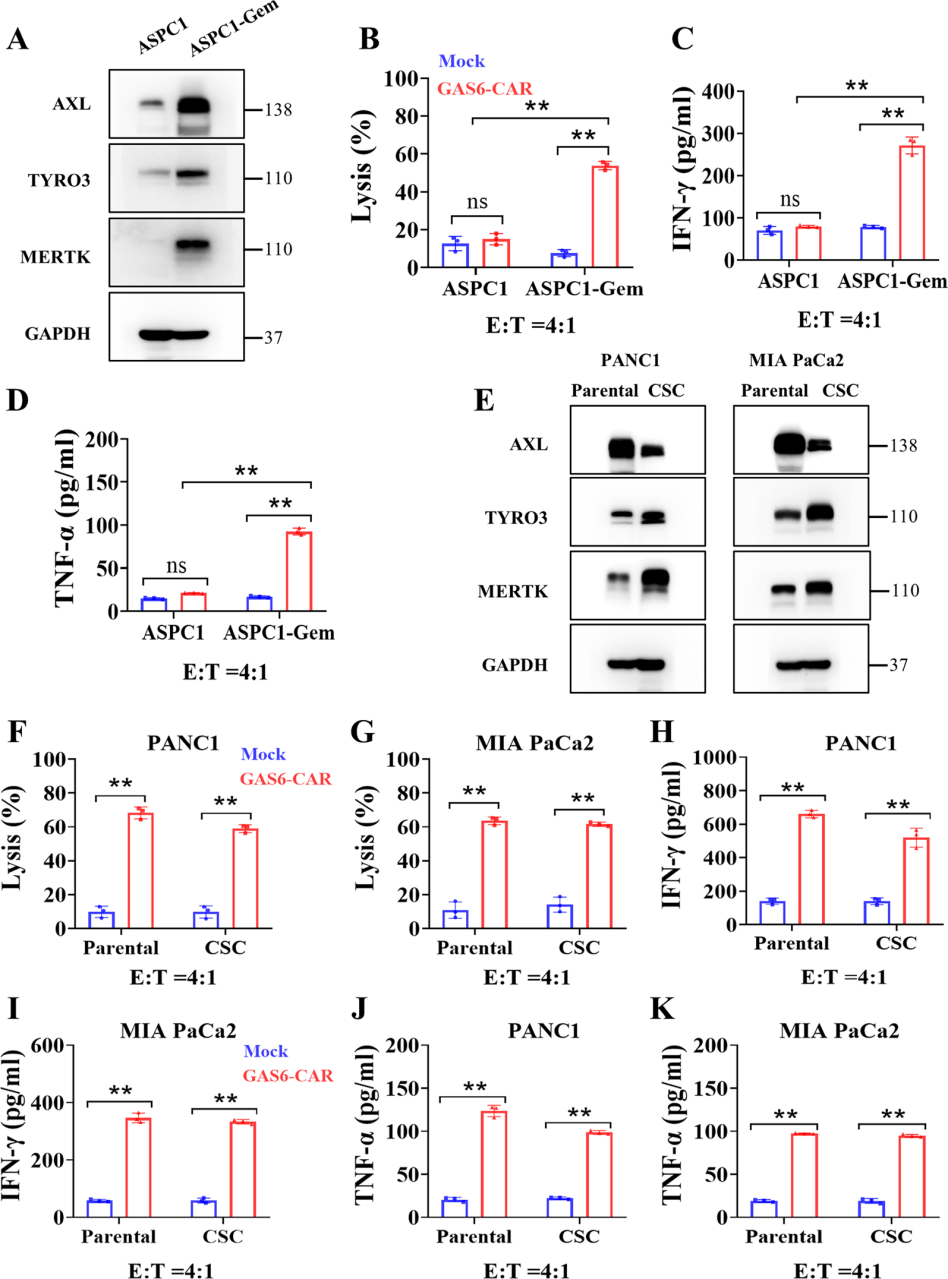
GAS6-CAR-T cells efficiently kill drug-resistant cells and cancer stem-like cells. **A** The levels of TAM proteins in ASPC1 and ASPC1-gemcitabine-resistant (ASPC1-Gem) cells were determined by western blot with GAPDH used as a loading control. **B** The cytotoxicity of GAS6-CAR-T cells on target cells was assessed an E/T ratio of 4:1 for 24 h (*n* = 3). ELISAs were used to detect IFN-γ (**C**) and TNF-α (**D**) release by T cells in coculture supernatants. **E** The levels of TAM proteins in parental cells (PANC1, MIA PaCa2) and cell line-derived cancer stem cells (PANC1-CSC and MIA PaCa2-CSC) were determined by western blot with GAPDH used as a loading control. The cytotoxicity of GAS6-CAR-T cells on luciferase-expressing target cells PANC1-CSC (**F**) and MIA PaCa2-CSC (**G**) was quantified at an E/T ratio of 4:1 for 24 h (*n* = 3). ELISAs were used to detect IFN-γ (**H**&** I**) and TNF-α (**J**&**K**) release by T cells in coculture supernatants

Drug resistance is a major factor underlying the failure of cancer treatments, and high levels of TAM proteins are strongly correlated with acquired drug resistance [16]. Therefore, we tested the effectiveness of GAS6-CAR-T cells in killing drug-resistant cell lines. First, we analyzed TAM proteins levels in ASPC1 and ASPC1-gemcitabine-resistant (ASPC1-Gem) pancreatic cancer cell lines. As shown in Fig. 1A and Fig. 4A, TAM proteins were prominent in ASPC1-Gem cells compared to the parental TAM-low cell line ASPC1. We next incubated luciferase-labeled ASPC1 and ASPC1-Gem cells with GAS6-CAR-T cells. CAR-T cells incubated with ASPC1-Gem cells showed a significant lysis activity (Fig. 4B), displayed increased mRNA expression levels for IFN-γ, TNF-α, IL-2, and IL-10 (Additional file 1: Fig. S4), and presented significantly elevated release of IFN-γ and TNF-α cytokines (Fig. 4C, D). Thus, GAS6-CAR-T cells can effectively target pancreatic cancer cell with gemcitabine resistance induced by TAM overexpression.

Cancer stem-like cells (CSCs) are important for tumor initiation, recurrence, metastasis, and major drivers of drug resistance [24, 25]. To test whether GAS6-CAR-T cells can also kill CSCs, we produced cancer cell line-derived CSCs as sphere cultures from PANC1 and MIA PaCa2 cells. The stemness of these CSCs was confirmed by the expression of stem cell markers CD133, CXCR4 and OCT4 (Additional file 1: Fig. S5). Interestingly, TYRO3 and MERTK in the CSCs were highly expressed, while AXL expression in the CSCs was unexpectedly decreased compared to the parental cell lines as detected by flow cytometry and WB (Fig. 1A and Fig. 4E). When incubated with GAS6-CAR-T cells, the viability of pancreatic CSCs was significantly reduced (Fig. 4F, G). This reduced viability was accompanied by increased expression of IFN-γ, TNF-α, and IL-2 (Additional file 1: Fig. S6) and release of IFN-γ **(**Fig. 4H, I) and TNF-α **(**Fig. 4J, K). To further validate the roles of individual TAM member expression in lysis, we evaluated the effects of knocking down individual TAM proteins. The silencing efficiency was tested by western blot (Additional file 1: Fig. S7A). Both AXL-shRNAs slightly reduced the cytotoxic effects of GAS6-CAR-T cells on CSCs (Additional file 1: Fig. S7B, C), while shRNAs targeting TYRO3 and MERTK significantly reduced the cytotoxic effects of GAS6-CAR-T cells (Additional file 1: Fig. S7B, C) and the secretion of IFN-γ (Additional file 1: Fig. S7D, E) and TNF-α (Additional file 1: Fig. S7F, G). These data suggest that GAS6-CAR-T cells induce significant cytotoxicity in pancreatic cancer stem-like cells that may attribute more to increased TYRO3 and MERTK.

### GAS6-CAR-T cells effectively and persistently inhibit the growth of cell line-derived xenograft

**Fig. 5:**
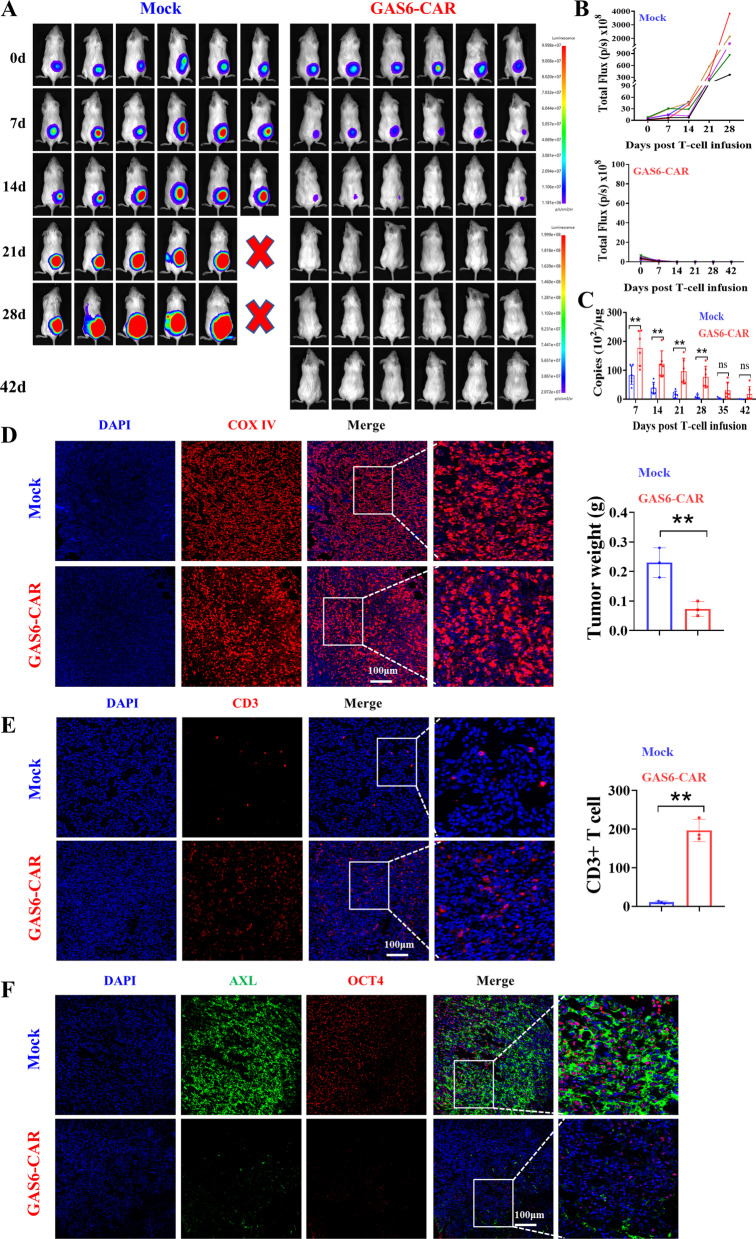
Antitumor effects of GAS6-CAR-T cells are analyzed in vivo. **A** Photographs of NCG mice subcutaneously injected with 5 × 10^5^ PANC1 cells; after receiving CAR-T-cell treatment, tumor volumes were monitored with bioluminescence at the indicated times. **B** Quantification of tumor bioluminescence levels (*n* = 5–6). **C** DNA copies of Mock T cells and GAS6-CAR-T cells in peripheral blood of mice were determined by real-time PCR. The expression of target genes was normalized GAPDH. 50 μL of venous blood collected from tail veins was collected after (days 7, 14, 21, 28, 35, 42) injecting GAS6-CAR-T cells into mice (*n* = 3–6). **D** Immunohistochemistry analysis of COX IV in tumor (*n* = 3), and the tumor weight at day 5 post-CAR-T cells infusion (the right). NCG mice subcutaneously injected with 5 × 10^5^ PANC1 cells received an infusion of CAR-T cells (1 × 10^7^ cells/mouse) at day 7, and the tumors were harvested after 5 days. **E** Immunohistochemistry analysis of CD3 in tumor (*n* = 3), and CD3 + T-cell numbers were counted in five randomly captured pictures of each mouse by ImageJ. **F** Co-immunofluorescence staining of AXL (green) and OCT4 (red) (*n* = 3)

To test the effects of GAS6-CAR-T cells on tumor growth in vivo, we established a xenograft mouse model by injecting PANC1 cells subcutaneously. Compared to Mock T cells, GAS6-CAR-T cells presented with significantly reduced tumor growth. Moreover, these mice remained completely tumor-free from day 21 after GAS6-CAR-T-cell treatment (Fig. 5A, B).

The copy number of CAR-T-cell DNA in peripheral blood was associated with tumor clearance, and copy numbers remained high for at least 42 days in the GAS6-CAR group compared to Mock controls (Fig. 5C). Previous research has shown that the number of infiltrating T cells within a tumor is closely related to antitumor activity [26]. The human origin of cells in tumor was determined by immunohistochemistry analysis with a commercially available antibody specific for the human mitochondrial marker COX IV [27], and the tumor weight was significantly reduced at day 5 after GAS6-CAR-T cells infusion (Fig. 5D), and increase in CD3 + T-cell infiltration in mice receiving GAS6-CAR-T cells compared to Mock-treated mice (Fig. 5E). These results suggest that GAS6-CAR-T cells can effectively home to target sites and inhibit the growth of tumor xenografts.

Double staining of AXL and OCT4 revealed that OCT4-positive stem cells are significantly reduced in GAS6-CAR-T group (Fig. 5F), thereby indicating that GAS6-CAR-T can target CSCs in vivo to enhance tumor clearance in consistent with the in vitro effects on CSCs.

### GAS6-CAR-T cells show antitumor activity in patient-derived xenografts

**Fig. 6:**
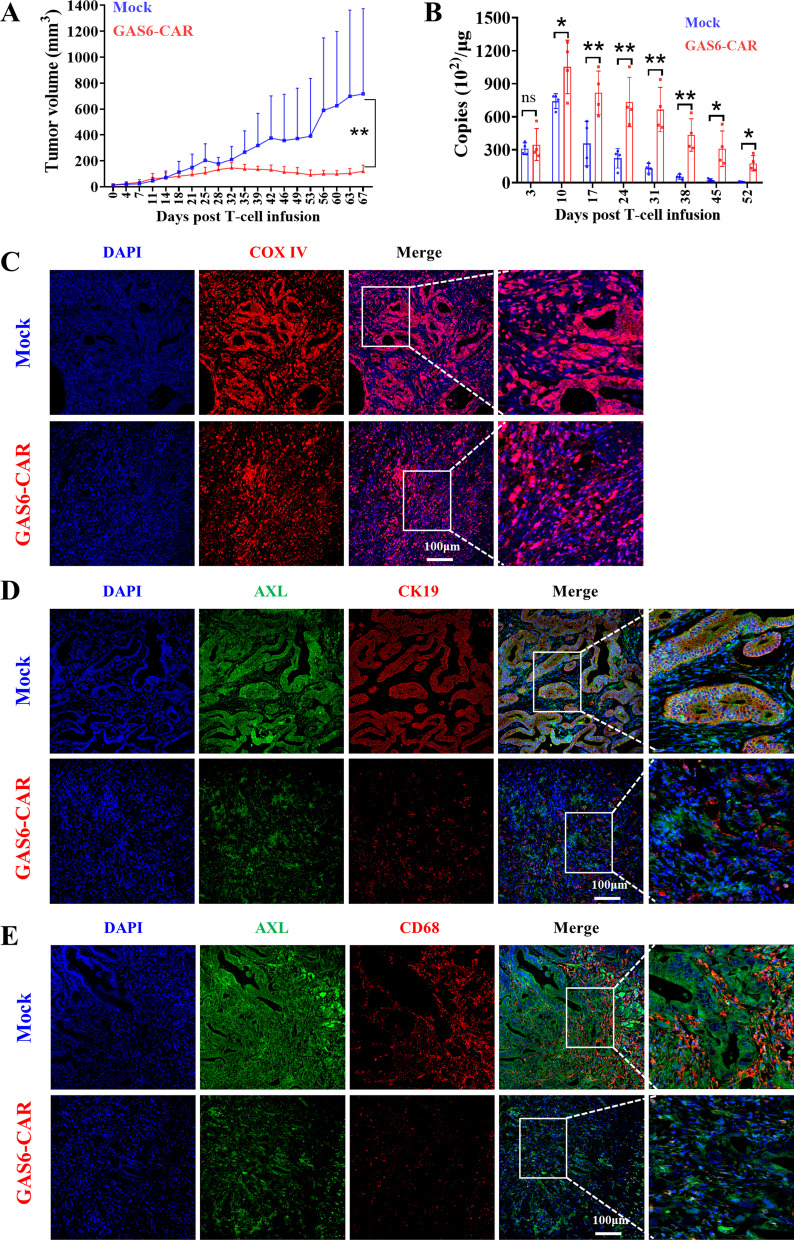
Cytotoxicity of GAS6-CAR-T cells on pancreatic cancer patient-derived in vivo xenograft model. **A** Tumor volumes were monitored at the indicated time points (*n* = 3–4). NCG mice were subcutaneously implanted with pancreatic tumor tissues and received an infusion of T cells (1 × 10^7^ cells/mouse) at days 14 and 23. **B** DNA copies of Mock T cells and GAS6-CAR-T cells in peripheral blood of mice were determined by real-time PCR. The expression of target genes was normalized GAPDH. 50 μL of venous blood collected from tail veins was collected after (days 3, 10, 17, 24, 31, 38, 45, 52) injecting GAS6-CAR-T cells into mice (*n* = 3–4). **C** Immunohistochemistry analysis of COX IV in tumor (*n* = 3). **D** Co-immunofluorescence staining of AXL (green) and CK19 (red) was used to test the cytotoxicity of CAR-T cells against AXL-positive tumor cells (*n* = 3). **E** Co-immunofluorescence staining of AXL (green) and CD68 (red) was used to test cytotoxic activity of CAR-T cells against AXL-positive tumor-associated macrophages (*n* = 3)

TAM proteins are also highly expressed in M2 macrophages that are the critical cell type in tumor microenvironment [28, 29]. And CD68 is a well-established marker for human macrophages [30]. PDX models have a striking advantage that non-tumor cells in tumor mass are of human origin. Therefore, we choose the pancreatic cancer patient-derived xenograft (PDX) models with the high expression of AXL in both cancer cell and macrophage to test whether GAS6-CAR-T cells can also eliminate cancer cells and macrophages in vivo. The patient-derived pancreatic tumor tissue was chosen according to the expression of markers of tumor cells (CK19) and macrophages (CD68), and AXL detected by immunofluorescence. An AXL-positive sample with a number of macrophages was chosen for xenograft experiment (Additional file 1: Fig. S8A, B). The clinical and pathologic characteristics of the patient are shown in Additional file 1: Table S3. As shown in Fig. 6A, intravenous administration of GAS6-CAR-T cells led to a significant suppression of pancreatic cancer PDX tumor growth, while tumors in the Mock group continued to grow rapidly. Also, the copy number of T-cell DNA in GAS6-CAR group was significantly higher than that in Mock group (Fig. 6B). In addition, GAS6-CAR-T cells could significantly reduce AXL and CK19 double-positive tumor cells (Fig. 6C, D), as well as AXL and CD68 double-positive macrophages (Fig. 6C, E). Overall, these data further strengthen the notion that GAS6-CAR-T cells may be an effective therapy targeting both tumor cells and tumor-associated macrophages.

### GAS6-CAR-T-cell treatment does not show overt side effects

**Fig. 7:**
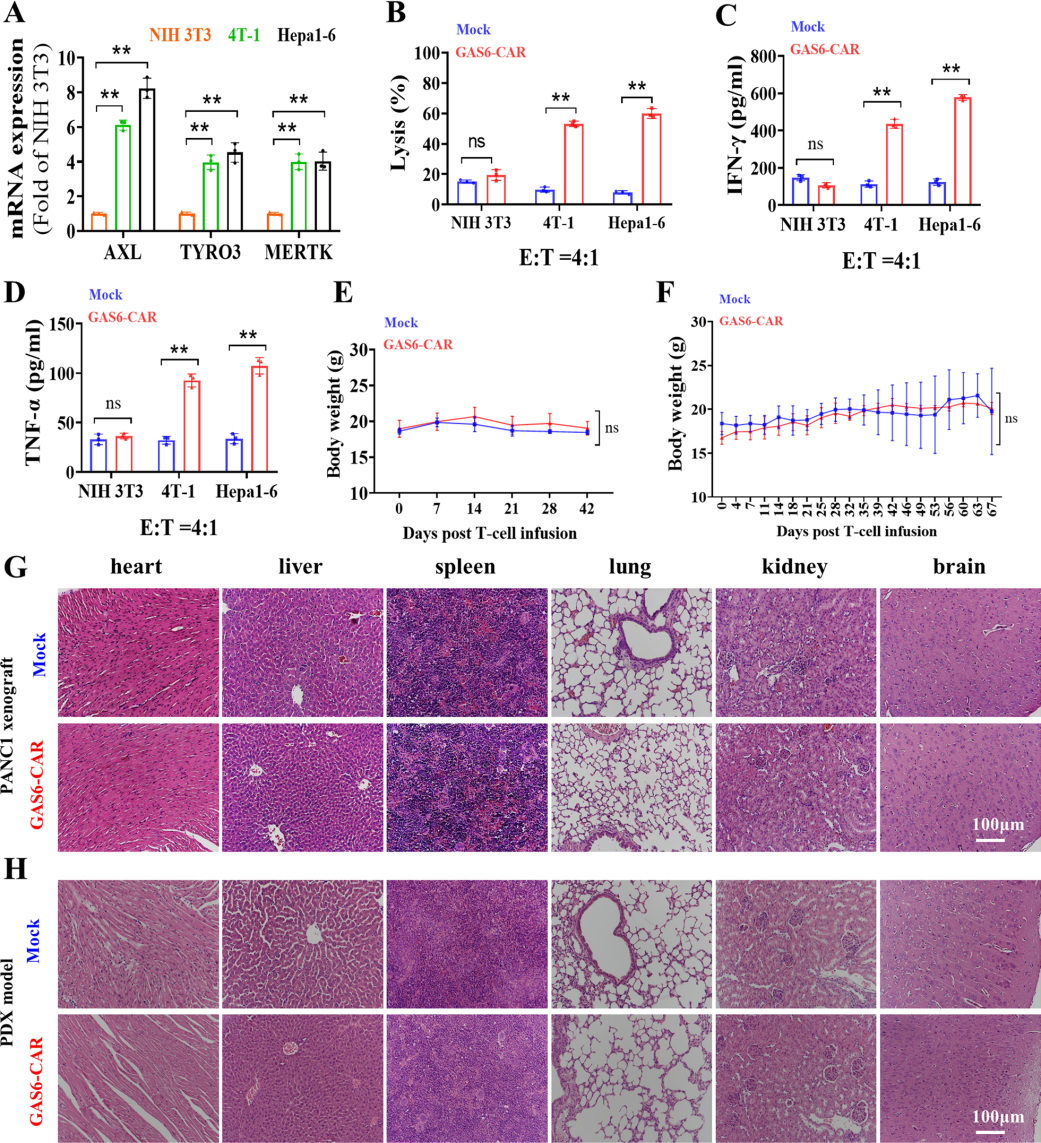
Safety of GAS6-CAR-T cells in mice. **A** PCR analysis of TAM expression in a non-cancerous mouse cell line (NIH 3T3) and mouse tumor cell lines (4 T-1, Hepa1-6). **B** The cytotoxicity of GAS6-CAR-T cells on mouse cell lines at an E/T ratio of 4:1 for 24 h (*n* = 3). ELISA-based quantification of T-cell-induced IFN-γ (**C**) and TNF-α (**D**) release in the culture supernatants. Body weights in PANC1 xenograft mice (*n* = 3–6) (**E**) and PDX model (*n* = 3–4) (**F**) after receiving CAR-T cells. Pathological analysis of the indicated organs following hematoxylin and eosin staining at the experimental endpoint of PANC1 xenograft (**G**) and PDX model (**H**)

GAS6-CAR-T cells did not lyse TAM-low ASPC1 and BxPC3 cell lines, suggesting it may spare normal tissues expressing lower AXL relative to cancer tissues. Due to the high conservation of receptor-ligand systems across species, human GAS6 could recognize the mouse TAM proteins at a similar level to human TAM proteins [31]. Therefore, we examined the effects of GAS6-CAR-T cells on murine tumor cell lines. GAS6-CAR-T cells effectively killed the high TAM-expressing mouse hepatoma carcinoma Hepa1-6 and breast cancer 4 T-1 cell lines (Fig. 7A, B) and induced the secretion of IFN-γ (Fig. 7C) and TNF-α (Fig. 7D), while GAS6-CAR-T cells displayed no significant toxicity or induction of cytokine release when exposed to the mouse embryonic fibroblast cell line NIH 3T3, which display low levels of TAM proteins (Fig. 7A–D). However, the mice of PANC1 xenograft and PDX model receiving GAS6-CAR-T cells did not show any obvious side effects, and they remained active and maintained similar body weights to the control group **(**Fig. 7E, F)**.** Furthermore, while large numbers of CD3 + T cells clustered in the tumor tissues of mice receiving GAS6-CAR-T cells, minimal numbers were observed in the major organs (Additional file 1: Fig. S9A), and no significant tissue damage or structural changes were observed in the major organs (heart, liver, spleen, lung, kidney, and brain) at day 5 or 42 of PANC1 xenograft (Additional file 1: Fig. S9B and Fig. 7G), and PDX model (Fig. 7H) after T-cell treatment in either group. These results suggest GAS6-CAR-T cells have minimal on-target off-tumor effects in vivo.

**Fig. 8:**
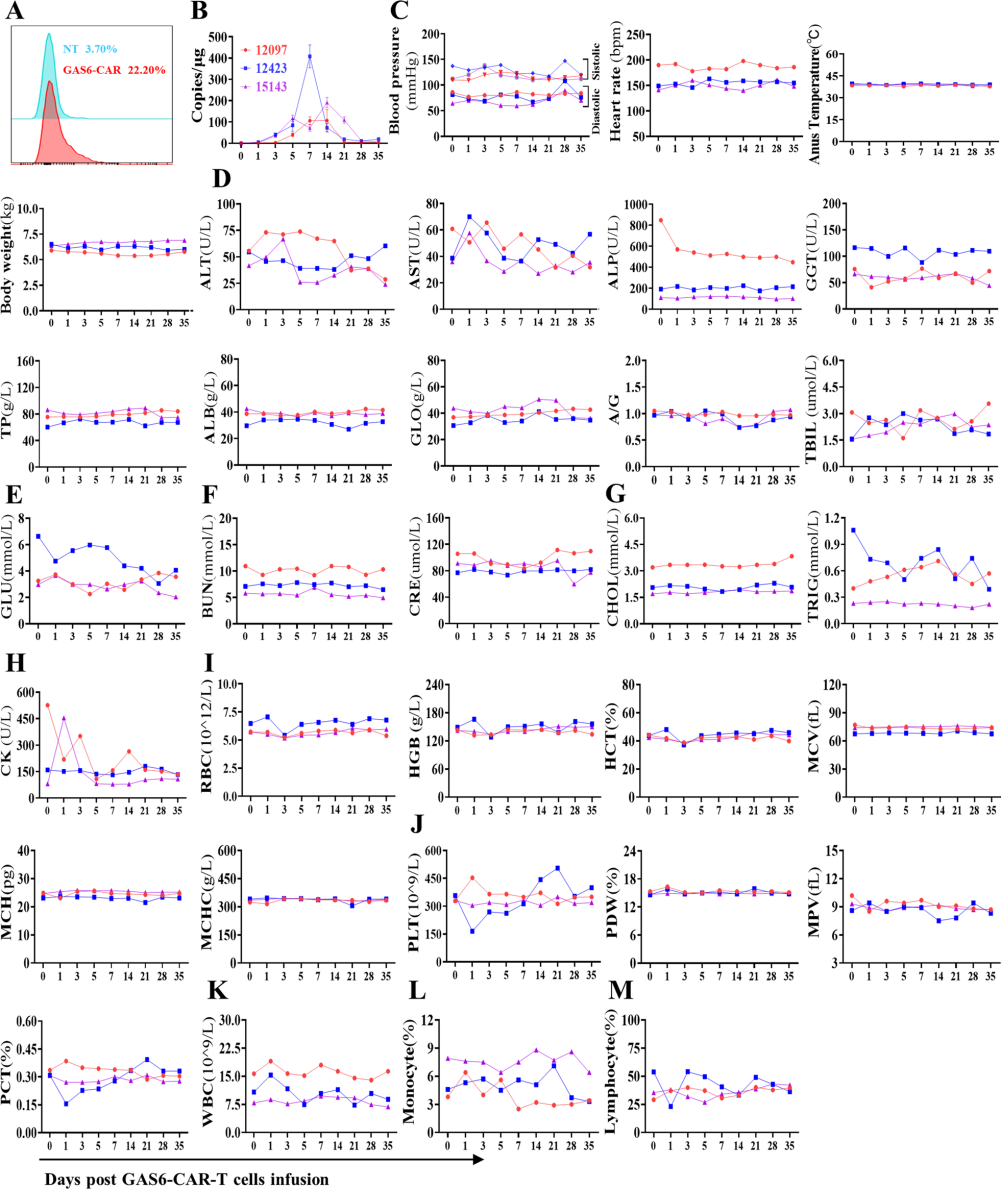
Safety of GAS6-CAR-T cells in nonhuman primate. **A** Transduction efficiency of GAS6-CAR into macaques T cells and non-transduced T (NT) cells was used as control. **B** DNA copies of GAS6-CAR-T cells in the peripheral blood of macaques were quantified by real-time PCR. GAS6-CAR-T cells (2 × 10^6^ cells/kg) were autologously infused to rhesus macaques, and the peripheral blood was collected before (day 0) and after (days 1, 3, 5, 7, 14, 21, 28, and 35) injecting GAS6-CAR-T cells into rhesus macaques (*n* = 3). **C** Analysis of physiological indexes (diastolic and systolic blood pressure, heart rate, anus temperature, body weight). Biochemical indicators for **D** alanine transaminase (ALT), aspartate aminotransferase (AST), alkaline phosphatase (ALP), γ-glutamine acylase (GGT), total protein (TP), albumin (ALB), globulin (GLO), ALB/GLO (A/G), total bilirubin (TBIL), **E** blood glucose (GLU), **F** blood urea nitrogen (BUN) and creatinine (CRE), **G** cholesterol (CHOL) and triacylglycerol (TRIG), **H** creatine kinase (CK) were tested. Blood routines for **I** red blood cell (RBC), hemoglobin (HGB), hematocrit (HCT), mean corpuscular volume (MCV), mean corpuscular hemoglobin (MCH), mean corpuscular hemoglobin concentration (MCHC), **J** platelet count (PLT), platelet distribution density (PDW), mean platelet volume (MPV), platelet volume ratio (PCT), **K** white blood cell (WBC), **L** monocyte, and **M** lymphocyte were analyzed

AXL protein (XP_028695606.1) of rhesus monkeys shares 96.16% homology with human AXL (NP_068713.2), and an AXL inhibitor in phase III clinical trial (AVB-S6-500) containing the extracellular domain of AXL to competitively bind GAS6 had the same affinity for GAS6 of human and cynomolgus monkeys, but not resulted in any obvious side effects in monkey [32]. To further evaluate the safety of CAR-T cells, GAS6-CAR-T cells prepared from rhesus macaques T cells were autologously infused (Fig. 8A). CAR-T cells show modest proliferation in vivo and persist about one month (Fig. 8B). As shown in Fig. 8C, there was no significant fluctuation in diastolic and systolic blood pressure, heart rate, anus temperature, and body weight on days 1, 3, 5, 7, 14, 21, 28, and 35 compared to day 0. GAS6-CAR-T cells did not result in significant changes of blood chemistry parameters, such as alanine transaminase (ALT), aspartate aminotransferase (AST), alkaline phosphatase (ALP), γ-glutamine acylase (GGT), total protein (TP), albumin (ALB), globulin (GLO), ALB/GLO (A/G), and total bilirubin (TBIL) for liver function (Fig. 8D); blood glucose (GLU) (Fig. 8E); blood urea nitrogen (BUN) and creatinine (CRE) for kidney function (Fig. 8F); cholesterol (CHOL) and triacylglycerol (TRIG) for serum lipids (Fig. 8G); and creatine kinase (CK) for cardiac function (Fig. 8H)**.** Also, the related blood indicators for red blood cells (RBC) (hemoglobin (HGB), hematocrit (HCT), mean corpuscular volume (MCV), mean corpuscular hemoglobin (MCH), mean corpuscular hemoglobin concentration (MCHC)) (Fig. 8I); platelets (platelet count (PLT), platelet distribution density (PDW), mean platelet volume (MPV), platelet volume ratio (PCT)) (Fig. 8J); white blood cell (WBC) (Fig. 8K); monocyte (Fig. 8L) and lymphocyte (Fig. 8M) did not show any obvious changes. These results suggest GAS6-CAR-T cells show high safety in vivo.

## Discussion

A limitation for the development of CAR-T therapies has been in the identification of ‘gold-standard’ tumor antigens, as it has been assumed that such antigens should be specifically expressed on tumors, but not on normal cells. Studies have reported that TAM proteins broadly express in not only numerous tumors, but also normal tissues or cells with basal level [5, 22, 33, 34]. While these findings may invoke safety concern on the use of GAS6-CAR-T cells, the affinity between natural ligands and receptors is usually lower than that between antigens and antibodies, and natural ligands-based CAR will probably efficiently attack tumor cells with higher target expression, but spare normal cells with lower target expression [35]. And we found no overt side effects and pathological changes to the major organs in mice. Furthermore, GAS6-CAR-T did not result in any obvious side effects on the physiological and biochemical indexes and blood routines of rhesus macaques. Thus, we believe that our results provide potent supports for the safety of GAS6-CAR-T cells.

CAR-T immunotherapy has achieved great success in treating hematological tumors. However, some patients experience relapse largely due to a loss of CAR-specific antigens on tumor cells or an exhaustion of CAR-T cells [36]. Resistance to CAR-T therapy due to antigen escape can be prevented by targeting multiple tumor markers using bi- or tri-specific CARs comprising two or three single-chain variable fragments. Bi-specific CARs that have shown to be effective anti-cancer agents include those targeting CD70-B7-H3 for several solid tumors [37], those targeting CD5–CD7 for leukemia [38], CD19–CD20 or CD19–CD22 for B-cell malignancies [39, 40]. Tri-specific CAR targeting CD19–CD20–CD22 has been shown to effectively inhibit the progression of B-cell tumors by reducing antigen escape [41]. Due to the ability of some natural ligands to bind multiple receptors, some natural ligand-based CAR-T cells can avoid tumor escape by targeting multiple targets [42]. A proliferation-inducing ligand (APRIL)-based CAR against BCMA and TACI inhibits the development of multiple myeloma [43], and B-cell-activating factor (BAFF) ligand-based CAR against BAFF-R, BCMA, and TACI inhibits the progression of B-cell tumors [36] by reducing antigen escape. As GAS6 is a key tumor cell survival factor and a common ligand for AXL, MERTK, and TYRO3 [3], our data imply that GAS6-CAR-T cells can recognize and kill cells overexpressing any of the TAM proteins, and ability to kill CSCs is more dependent on the higher expression of TYRO3 and MERTK than AXL in contrast to parental cell lines. Thus, GAS6-CAR-T cells may provide enhanced antitumor effects by limiting antigen escape.

Resistance to cancer drug treatment represents the most common cause of cancer deaths [44]. It is acquired through multiple avenues, such as acquired resistance to chemotherapies and CSCs [24, 44]. TAM proteins are also known to promote acquired resistance to chemotherapies and CSCs [16, 45]. We demonstrate CSCs express a higher level of TYPO3 and MERTK compared to parental cell lines and the TAM expression pattern can also be effectively targeted by GAS6-CAR-T cells. Upregulated AXL expression in gemcitabine-resistant ASPC1 cells results in lysis by GAS6-CAR-T cells in comparison with little effects on AXL-low ASPC1 cells. These suggest GAS6-CAR-T cells are ideal to be combined with conventional chemotherapies and may therefore overcome the drug resistance.

The pancreatic cancer microenvironment is characterized by extremely dense connective tissue and highly immunosuppressed cells, with non-tumor cell components comprising up to 90% of the total tumor mass [46]. Tumor-associated macrophages as the main immunosuppressive cells in the microenvironment enhance immune suppression and angiogenesis, secrete inhibitory cytokines, and increase the carcinogenic ability of CSCs and their resistance to chemotherapy [47]. The immunosuppression caused by tumor-associated macrophages is a significant barrier for effective pancreatic cancer therapy [28]. Recently, CAR-T cells targeting tumor-associated macrophages were shown to be an effective strategy for slowing tumor progression [48]. Moreover, CAR-T cells targeting F4/80 [49] or CD123 [50] can kill M2-type macrophages in the microenvironment and delay tumor growth. In addition to the roles in tumor cell, TAM proteins also participate in the polarization of M1 macrophages to M2 macrophages and overexpress in tumor-associated macrophages, and targeting TAM receptors can also effectively inhibit the function of macrophages and eliminate tumor cells [29]. We demonstrated that GAS6-CAR-T cells can inhibit the growth of pancreatic cancer PDX models by elimination of both AXL-positive tumor cells and tumor-associated macrophages. Therefore, it is expected that GAS6-CAR-T cells probably offer better clinical outcomes by targeting both tumor cells and tumor-associated macrophages.

## Conclusions

Here, we have shown that GAS6-based CAR-T cells can effectively kill TAM-positive pancreatic tumor cells and inhibit the growth of xenograft tumors in vivo by eliminating both tumor cells and tumor-associated macrophages. GAS6-CAR-T cells were also demonstrated to recognize mouse TAM and kill mouse tumor cell lines, but did not cause any significant side effects in xenograft mice. And GAS6-CAR-T cells also did not show any significant side effects on nonhuman primate. These suggest that GAS6-based CAR-T cells can be a promising and safe therapeutic strategy for pancreatic cancer.

## Supplementary Information

**Additional file 1**.

## Data Availability

The data generated in this study are available within the article and its supplementary data files.

## Abbreviations

CAR-T: Chimeric antigen receptor T
GAS6: Growth arrest-specific protein 6
ASPC1-Gem: ASPC1-gemcitabine resistant
NT: Non-transduced T cells
CSCs: Cancer stem-like cells
PDX: Patient-derived xenograft
ALT: Alanine transaminase
AST: Aspartate aminotransferase
ALP: Alkaline phosphatase
GGT: γ-Glutamine acylase
TP: Total protein
ALB: Albumin
GLO: Globulin
A/G: ALB/GLO
TBIL: Total bilirubin
BUN: Blood urea nitrogen
CRE: Creatinine
CHOL: Cholesterol
TRIG: Triacylglycerol
CK: Creatine kinase
RBC: Red blood cells
HGB: Hemoglobin
HCT: Hematocrit
MCV: Mean corpuscular volume
MCH: Mean corpuscular hemoglobin
MCHC: Mean corpuscular hemoglobin concentration
PLT: Platelet count
MPV: Mean platelet volume
PCT: Platelet volume ratio
PDW: Platelet distribution density
WBC: White blood cell
ELISA: Enzyme-linked immunosorbent assay
E/T: Effector to target
IHC: Immunohistochemistry
IF: Immunofluorescence
WB: Western blot
FC: Flow cytometry
