# Supplementary material for: GAS6-based CAR-T cells exhibit potent antitumor activity against pancreatic cancer
Source: J Hematol Oncol. 2023 Jul 20;16:77. doi: 10.1186/s13045-023-01467-9 (PMC10357739; doi:10.1186/s13045-023-01467-9)
Supplement: Supplementary file 1 — Additional file 1. [file 13045_2023_1467_MOESM1_ESM.docx]

**Supplementary Materials for**

**GAS6-based CAR-T cells exhibit potent antitumor activity against**

**pancreatic cancer**

Jiawei Fan^1†^, Ye Yu^1†^, Lanzhen Yan^1†^, Yuncang Yuan^1^, Bin Sun^1^, Dong Yang^1^, Nan Liu^1^,

Jing Guo^1^, Jie Zhang^2*^ & Xudong Zhao^1*^

†These authors contributed equally to this work.

***Correspondence**: zjie@scu.edu.cn; zhaoxudong@wchscu.cn. Tel: 028-85164204.

^1^ Division of Abdominal Tumor Multimodality Treatment and Laboratory of Animal Tumor Models, Cancer Center and State Key Laboratory of Biotherapy and National Clinical Research Center for Geriatrics and Frontiers Science Center for Disease-related Molecular Network, West China Hospital, Sichuan University, Chengdu, 610041, Sichuan, China.

^2^ Core Facilities of West China Hospital, Sichuan University, Chengdu, 610041, Sichuan, China.

**This file includes: Figures S1 to S9, Tables S1 to S3.**


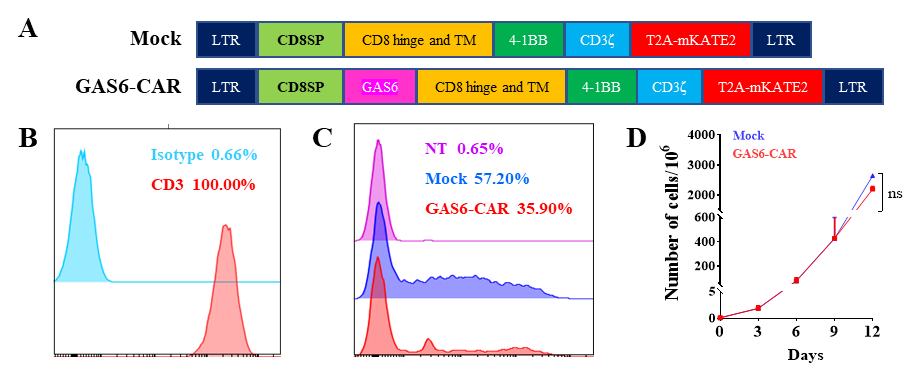


**Figure S1. Characteristics of GAS6-CAR.** **(A)** Schematic representation of Mock and GAS6-CAR. **(B)** Flow cytometry analysis of CD3 expression in primary human T cells. The percentages of stained cells (red) and isotype controls (blue) were highlighted. **(C)** Transduction efficiency of Mock and GAS6-CAR into T cells, non-transduced T (NT) cells was used as control. CAR expression was detected using the fusion protein T2A-mKATE2, and analyzed by flow cytometry on 3^rd^ day after transduction. Representative results were shown. **(D)** Accumulative growth curves of the CAR-T cells *in vitro*. Cell number was counted every 3 days (*n* = 3).

**
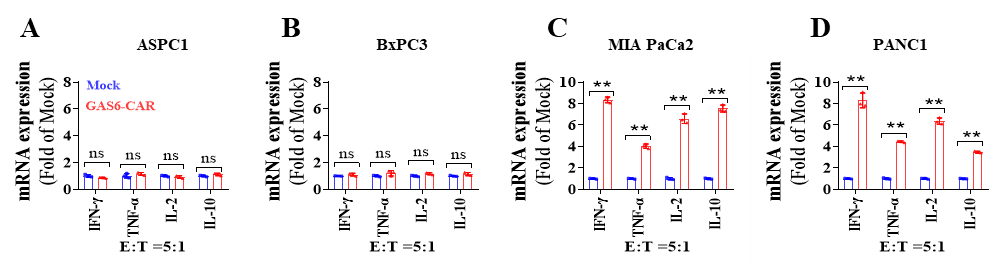
**

**Figure S2. Analysis of cytokine transcripts in CAR-T cells incubated with pancreatic cancer cell lines.** Mock and GAS6-CAR-T cells were incubated with pancreatic cancer cell lines at an E:T ratio of 5:1. After 24 hours of co-culture, T cells were harvested and RNA extracted to quantify the IFN-γ, TNF-α, IL-2 and IL-10 transcripts in ASPC1 **(A)**, BxPC3 **(B)**, MIA PaCa2 **(C)**, and PANC1**(D)** by real time PCR (*n* = 3).


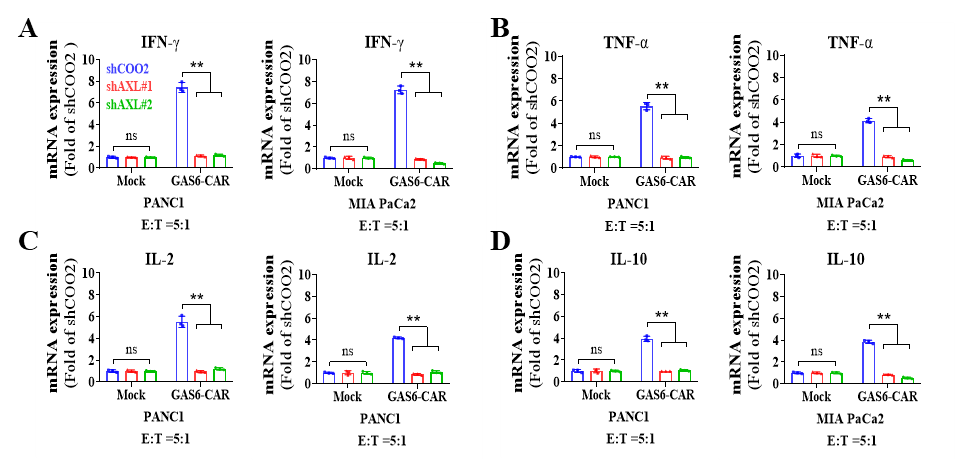


**Figure S3. Effects of AXL knockdown in target cells on cytokine genes expression in CAR-T cells.** Mock and GAS6-CAR-T cells were incubated with PANC1-shAXL and MIA PaCa2-shAXL cells at an E:T ratio of 5:1. After 24 hours of co-culture, T cells were harvested and RNA extracted to quantify IFN-γ **(A)**, TNF-α **(B)**, IL-2 **(C)**, and IL-10 **(D)** transcripts by real time PCR (*n* = 3).


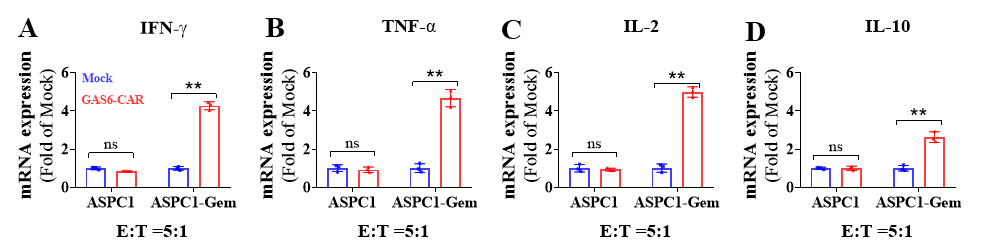


**Figure S4. Analysis of cytokine genes expression in CAR-T cells incubated with drug-resistant pancreatic cancer cell lines.** Mock and GAS6-CAR-T cells were incubated with ASPC1 and ASPC1-Gem cells at an E:T ratio of 5:1. After 24 hours of co-culture, T cells were harvested and RNA extracted to quantify the IFN-γ **(A)**, TNF-α **(B)**, IL-2 **(C)**, and IL-10 **(D)** transcripts by real time PCR (*n* = 3).


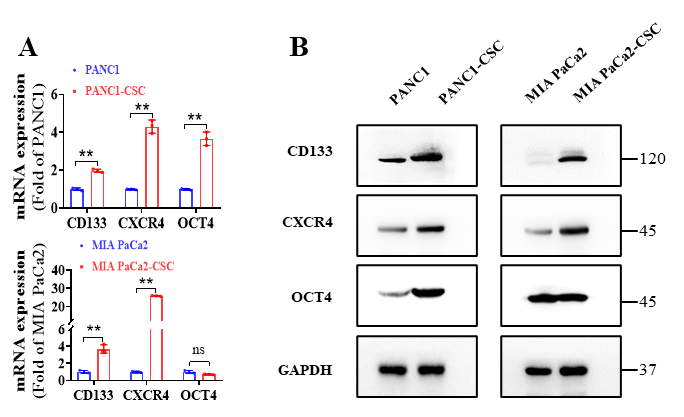


**Figure S5. Identification of pancreatic cancer stem-like cells. (A)** Quantification of RNA transcripts of CD133, CXCR4, and OCT4 by real time PCR (*n* = 3). **(B)** Protein levels for CD133, CXCR4, and OCT4 in parental cells (PANC1, MIA PaCa2) and cell line derived cancer stem-like cells (PANC1-CSC and MIA PaCa2-CSC) were detected by western blot. GAPDH was used as a loading control.


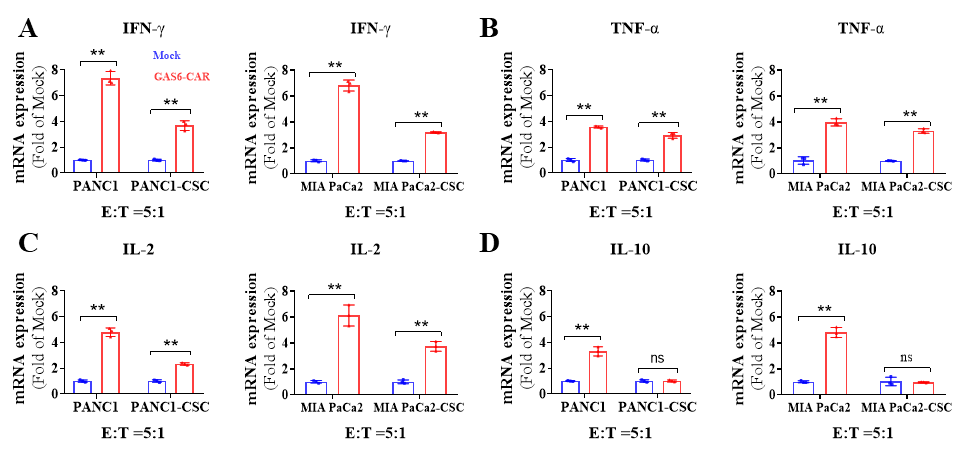


**Figure S6. Analysis of cytokine genes expression in CAR-T cells incubated with pancreatic cancer stem-like cells.** Mock and GAS6-CAR-T cells were incubated with parental cells (PANC1, MIA PaCa2) and cell line derived cancer stem-like cells (PANC1-CSC and MIA PaCa2-CSC) at an E:T ratio of 5:1. After 24 hours of co-culture, T cells were harvested and RNA extracted to quantify the IFN-γ **(A)**, TNF-α **(B)**, IL-2 **(C)**, and IL-10 **(D)** transcripts by real time PCR (*n* = 3).

**
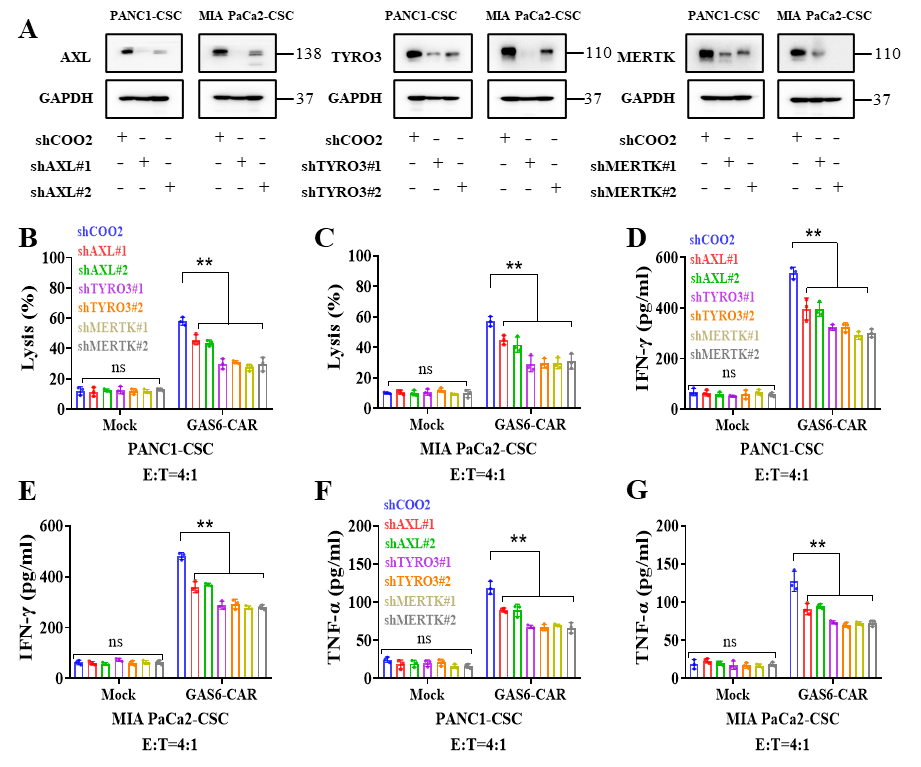
**

**Figure S7. The cytotoxicity of GAS6-CAR-T cells on CSCs attributes to TYRO3 and MERTK. (A)** The knockdown efficiency of shRNAs targeting AXL, TYRO3, or MERTK in PANC1-CSC and MIA PaCa2-CSC was tested by western blot, with GAPDH used as a loading control. Cytotoxicity of GAS6-CAR-T cells on target cells PANC1-CSC-shRNA **(B)** and MIA PaCa2-CSC-shRNA **(C)** was tested at an E:T ratio of 4:1 for 24 hours (*n* =3). ELISA-based quantification of IFN-γ **(D-E)** and TNF-α **(F-G)** release in response to coculture with Mock or GAS6-CAR-T cells.


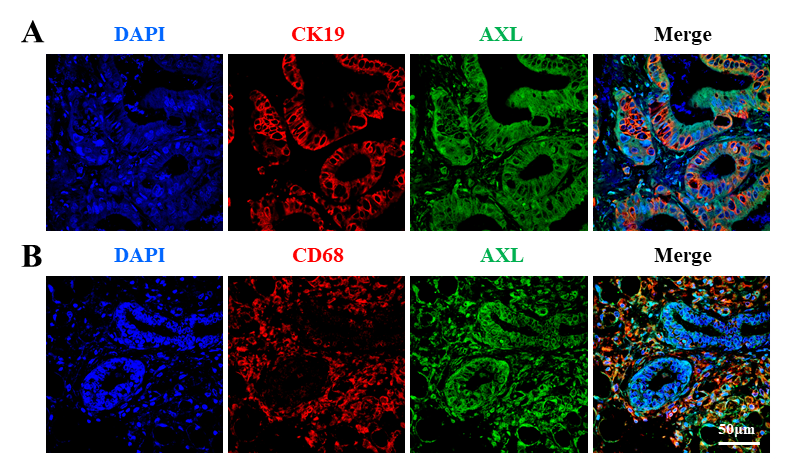


**Figure S8. The immunohistochemistry of patient-derived pancreatic cancer specimens.** Representative confocal microscopy images of immunofluorescence staining for pancreatic cancer cell marker CK19 (red) **(A)** and macrophages marker CD68 (red) **(B)** with AXL (green).

**
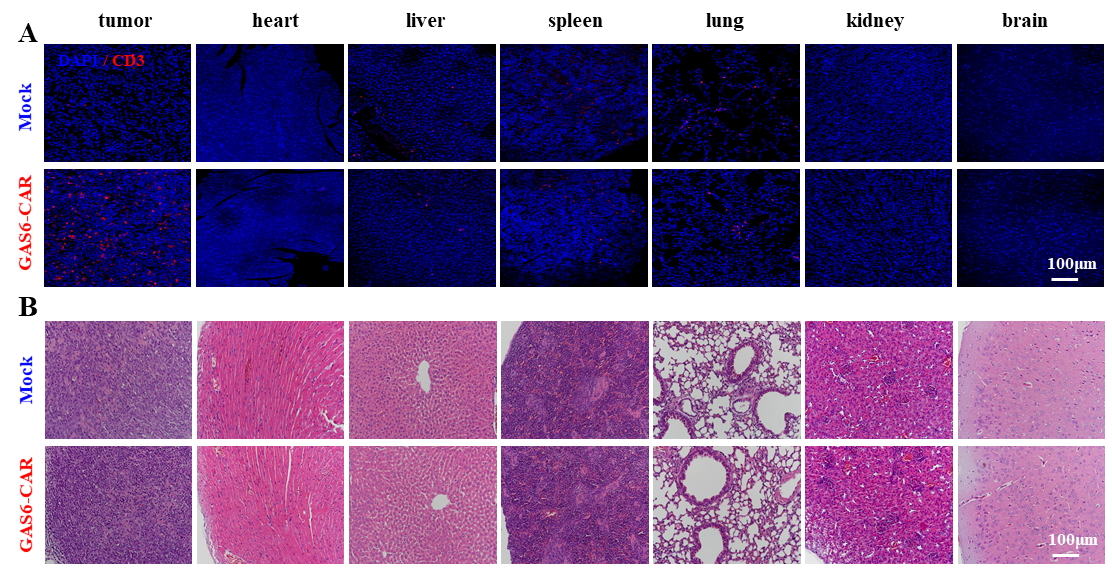
**

**Figure S9. GAS6-CAR-T cells does not cause evident toxicity in mice. (A)** Representative confocal microscopy images of CD3+ T cells in tumors and major organs (*n* = 3 mice per group). NCG mice were subcutaneously injected with 5×10^5^ PANC1 cells, and received an infusion of T cells (1×10^7^ cells/mouse) at day 7, then the tumor and major organs were harvested after 5 days. **(B)** Representative micrographs of major organs stained with hematoxylin and eosin (*n =* 3 mice per group).

| **Antibody** | **Company** | **Art. No.** | **Apply (dilution)** |
| --- | --- | --- | --- |
| **GAPDH** | Cell Signaling Technology | 2118 | WB (1:5000) |
| **AXL** | Cell Signaling Technology | 8661 | WB (1:2000)  IHC (1:200)  FC (1: 1000) |
| **TYRO3** | Cell Signaling Technology | 5585 | WB (1:1000)  FC (1: 1000) |
| **COX IV** | Cell Signaling Technology | 3E11 | IHC (1:1000) |
| **MERTK** | Santa Cruz | sc-365499 | WB (1:1000)  FC (1: 500) |
| **CD133** | Santa Cruz | sc-365537 | WB (1:500) |
| **OCT4** | Santa Cruz | sc-5279 | WB (1:1000)  IHC (1:100) |
| **CXCR4** | Santa Cruz | sc-53534 | WB (1:500) |
| **CK19** | Proteintech | 10712-1-AP | IHC (1:500) |
| **CD3** | HUABIO | ET1607-29 | IHC (1:200) |
| **CD68** | HUABIO | EM1706-11 | IHC (1:500) |
| **Mouse Anti-Human CD3** | BD Biosciences | 552127 | FC (1:1000) |
| **Anti-Rabbit IgG** | Sigma | A6154-1ML | WB (1:5000)  IHC (1:10000) |
| **Anti-Mouse IgG** | Sigma | A4416-1ML | WB (1:5000)  IHC (1:10000) |
| **Cy3 goat anti-Mouse IgG** | Invitrogen | A10521 | FC (1:1000) |
| **Cy3 goat anti-Rabbit IgG** | Invitrogen | A10520 | FC (1:1000) |

**Table S1. Antibodies** **used in the study**

| **Genes** | **Organism** | **Forward primer (5'→3')** | **Reverse primer (5'→3')** |
| --- | --- | --- | --- |
| **CD133** | Human | AGTCGGAAACTGGCAGATAGC | GGTAGTGTTGTACTGGGCCAAT |
| **CXCR4** | Human | AAACTGAGAAGCATGACGGACAA | GCCAACATAGACCACCTTTTCAG |
| **OCT4** | Human | GTGCCGTGAAGCTGGAGAA | TGGTCGTTTGGCTGAATACCTT |
| **AXL** | Human | CAGTGCCAAATCCGGGGAG | GCCTGCGTGCCCCTG |
| **TYRO3** | Human | CAGCCGGTGAAGCTCAACT | TGGCACACCTTCTACCGTGA |
| **MERTK** | Human | ACCTCTGTCGAATCAAAGCCC | CTGCACACTGGTTATGCTGAA |
| **AXL** | Mouse | ATGGCCGACATTGCCAGTG | CGGTAGTAATCCCCGTTGTAGA |
| **TYRO3** | Mouse | GCCTCCAAATTGCCCGTCA | CCAGCACTGGTACATGAGATCA |
| **MERTK** | Mouse | CAGGGCCTTTACCAGGGAGA | TGTGTGCTGGATGTGATCTTC |
| **IL2** | Human | ATGTACAGGATGCAACTCCTG | TCAAGTCAGTGTTGAGATGATGC  TTTGACAAAA |
| **IL10** | Human | AGGGCACCCAGTCTGAGAACA | CGGCCTTGCTCTTGTTTTCAC |
| **TNF-α** | Human | CTGGGCAGGTCTACTTTGGG | CTGGAGGCCCCAGTTTGAAT |
| **IFN-γ** | Human | TCGGTAACTGACTTGAATGTCCA | TCGCTTCCCTGTTTTAGCTGC |
| **18S rRNA** | Human/  Mouse | GTAACCCGTTGAACCCCATT | CCATCCAATCGGTAGTAGCG |
| **CD3** | Human | CCCCTTGCTCTGCTTCTTCA | CGCTCCTGCTGAACTTCACT |
| **GAPDH** | Human | CATCACTGCCACCCAGAAGACTG | ATGCCAGTGAGCTTCCCGTTCAG |

**Table S2.** **Primer sequences** **for real time PCR**

| **Parameters** | **Clinical characteristics** |
| --- | --- |
| **Sex/Age** | Female/55 |
| **Tumor grade** | Ⅱ |
| **Lymph node metastases** | NO |
| **Distant metastases** | NO |
| **Nerve invasion** | NO |
| **Vascular invasion** | NO |
| **Radiotherapy** | NO |
| **Chemical therapy** | NO |
| **Necrosis** | NO |
| **CDX2、MUC2** | NO |
| **CK7、CK19、CK20、CD68** | Yes |
| **DPC4、MUC1、Villin** | Yes |

**Table S3. Clinical and pathologic characteristics of original patient-derived pancreatic cancer specimens**
